# Reduction of T-Helper Cell Responses to Recall Antigen Mediated by Codelivery with Peptidoglycan *via* the Intestinal Nanomineral–Antigen Pathway

**DOI:** 10.3389/fimmu.2017.00284

**Published:** 2017-03-17

**Authors:** Rachel E. Hewitt, Jack Robertson, Carolin T. Haas, Laetitia C. Pele, Jonathan J. Powell

**Affiliations:** ^1^Department of Veterinary Medicine, University of Cambridge, Cambridge, UK; ^2^Medical Research Council, Department of Mineral Science and Technology, Elsie Widdowson Laboratory, Cambridge, UK

**Keywords:** nanomineral, T cells, intestinal tolerance, peptidoglycan, IL-10, antigen-presenting cells, programmed death receptor ligand 1

## Abstract

Naturally occurring intestinal nanomineral particles constituently form in the mammalian gut and trap luminal protein and microbial components. These cargo loaded nanominerals are actively scavenged by M cells of intestinal immune follicles, such as Peyer’s patches and are passed to antigen-presenting cells. Using peripheral blood mononuclear cell populations as an *in vitro* model of nanomineral uptake and antigen presentation, we show that monocytes avidly phagocytose nanomineral particles bearing antigen and peptidoglycan (PGN), and that the presence of PGN within particles downregulates their cell surface MHC class II and upregulates programmed death receptor ligand 1. Nanomineral delivery of antigen suppresses antigen-specific CD4^+^ T cell responses, an effect that is enhanced in the presence of PGN. Blocking the interleukin-10 receptor restores CD4^+^ T cell responses to antigen codelivered with PGN in nanomineral form. Using human intestinal specimens, we have shown that the *in vivo* nanomineral pathway operates in an interleukin-10 rich environment. Consequently, the delivery of a dual antigen–PGN cargo by endogenous nanomineral *in vivo* is likely to be important in the establishment of intestinal tolerance, while their synthetic mimetics present a potential delivery system for therapeutic applications targeting the modulation of Peyer’s patch T cell responses.

## Introduction

A purposeful role for endogenously formed nanomineral particles of the intestine, serving as natural transfection agents, was proposed over a decade ago, arising from studies on the influence of particulates in intestinal disease (1). Naturally forming intestinal nanominerals have recently been characterized as nano-sized amorphous magnesium-substituted calcium phosphate (AMCP) species, constituently formed through self-assembly in the milieu of the intestinal lumen (2). These particles comprise a blend of nanomineral, protein, and microbial components such as peptidoglycan (PGN). Self-assembly of intestinal nanomineral is conserved under significant calcium and phosphate dietary deficiency in mice, signifying that formation of particles in the intestine may occur for physiological benefit (2). The occurrence of nanomineral particles traversing specialized microfold “M” cells into intestinal lymphoid aggregates such as Peyer’s patches, coupled with their appearance within antigen-presenting cells (APCs), advocates an antigen “shuttling” function of these particles (2). *In situ* studies of intestinal lymphoid tissue have revealed that the immuno-inhibitory receptor, programmed death receptor ligand 1 (PD-L1), is heavily upregulated on cells that have received nanomineral-mediated delivery of PGN, suggesting that these nanomineral particles may have a role in the maintenance of intestinal tolerance toward gut derived antigen and microbiota in the healthy gut (2).

Microbial components, such as PGN, are recognized by cellular pattern-recognition receptors (PRRs), including toll-like receptors (TLRs) and NOD-like receptors (3). Nanoparticulate carriage of antigen, especially when combined with PRR ligands is normally associated with the enhancement of immune responses, and, therefore, nanoparticles have been broadly utilized in vaccine strategies (4–6). PRR recognition of microbial components triggers innate immune processes, but also facilitate the development of adaptive immune responses. This is enabled through the secretion of cytokines, the modulation of regulatory receptors (such as members of the B7 superfamily), and the stimulation of antigen processing and presentation by APCs (3, 7–9). As well as facilitating robust immune responses, the tolerance-inducing nature of some PRR ligands has also been established under certain circumstances (10–13). The expression of PRRs occurs in many cell types throughout the intestine, with numerous roles, including promoting the production of factors associated with tissue homeostasis, luminal sampling, and the development of specific cell subsets (14, 15). In the intestine, recognition of resident microbiota *via* PRRs appears a seminal feature in the maintenance of tolerance in the healthy gut, while failure in such processes may be involved in the development of inflammatory disease (14–18). The importance of PRRs in intestinal tolerance is emphasized by mouse studies, where negating TLR2, TLR4, or the corresponding signaling components results in aberrant immune responses and gut injury (14). Similarly in humans, defects in the bacterial sensing receptor NOD-2 are associated with the intestinal inflammatory disorder Crohn’s disease (CD), characterized as activated APC interacting with a dominant CD4^+^ Th1 lymphocyte population (19).

The recent discovery of endogenous intestinal nanominerals traversing into nearby lymphoid tissue, followed by uptake by APCs offers a novel, additional route by which luminal antigen and components of intestinal microbiota may be continually sampled. In the intestine, nanomineral AMCP particles were found to be colocalized with dietary antigen and PGN, a microbial component ubiquitous in the intestinal tract (2). Further *in vitro* studies using synthetic mimetics of endogenous AMCP particles (sAMCP) demonstrated a marked capacity of sAMCP to trap and deliver macromolecules which were then delivered to endosomal/lysosomal compartments upon uptake by APCs. Furthermore, the sAMCP construct itself failed to either significantly alter gene regulation of APCs in response to PGN challenge or to alone induce innate inflammatory responses (20). In this study, we establish APC responsiveness to protein antigen and PGN delivered as nanomineral cargo, as found in the intestine, and the subsequent influence on T helper cell responses. Synthetic AMCP was loaded with PGN and antigenic protein. PGN was chosen as the model PRR ligand due to its presence in the intestinal lumen, both as a component of the bacterial cell wall, particularly in Gram-positive bacteria, and as cell wall fragments released from commensal bacteria. By mimicking the attributes of intestinal nanomineral particles (20), we demonstrate *in vitro* suppression of antigen-specific CD4^+^ Th1 cell responses to cognate antigen thereby supporting a role for the nanomineral–antigen pathway in the control and maintenance of immune responsiveness in the gut and the use of nanomineral mimetics for the modulation of antigen-specific T cell responses.

## Materials and Methods

The study was approved by the local ethics committee; University of Cambridge, Human Biology Research Ethics Committee, application HBREC.2015.10.

### Particle Synthesis

Synthetic amorphous magnesium-substituted calcium phosphate (sAMCP) nanomineral particles were prepared as previously described (20). Briefly, for the preparation of particles incorporating PGN (*Staphylococcus aureus*, Fluka), PGN was added to phosphate (PO_4_) solution [containing 39 mM (NH_4_)HPO_4_ (Sigma-Aldrich) in 0.15 M TRIS buffer, adjusted to pH 8 with hydrochloric acid], prior to mixing in equal parts with calcium–magnesium–BSA solution (BSA; Sigma-Aldrich Company Ltd., Dorset, UK) dissolved in a Ca/Mg solution [containing 35 mM CaCl_2_ (Sigma-Aldrich), 7.2 mM MgCl_2_ (Sigma-Aldrich) in 0.15 M Tris buffer (Sigma-Aldrich)] at 1 mg/mL for a final PGN concentration of 50 μg/mL mixed solution. For particles incorporating protein purified derivative (PPD) of tuberculin (Statens Serum Institute, Denmark), PPD was added to the phosphate solution prior to mixing with the calcium–magnesium–BSA solution for a final PPD concentration of 100 μg/mL, which was then incubated with gentle rotation at room temperature for 1 h to allow particle formation. Particles were precipitated by centrifugation (1,500 rpm for 5 min), washed in pH 10 water, followed by washing in tissue culture grade water before re-suspending in tissue culture media at half of the original particle solution volume.

### Nanosight NS500 Analysis

Size distribution of amorphous calcium phosphate particles was examined using a Nanosight NS500 instrument and NTA 2.1 software (NanoSight Ltd.). Freshly synthesized particles were diluted 1:10 to an optimal particle concentration of between 10^7^ and 10^9^ particles/mL immediately prior to loading into the viewing chamber. Camera levels and focus were adjusted for optimal particle visualization and tracking. A capture duration of 90 s was used to record each video for NTA analysis. Mode values were obtained through area-under-curve analysis in GraphPad Prism 6.03 while average size and percentiles were determined in Microsoft Excel. For the latter, data were transformed into cumulative percentage and exact D10, D50, and D90 values calculated with linear interpolation using the two data points above and below the 10th, 50th, and 90th percentile, respectively.

### Quantification of Organic Matter within Particles

Following synthesis, empty particles or particles containing BSA, PPD, and PGN (alone or in conjunction) were centrifuged at 1,500 rpm (5 min) and supernatants were collected. After further washing twice, in pH 10 water, particles were dissolved in 100 mM citric acid buffer (pH 3) to release the organic material. Total protein content was measured using the Bradford protein assay (as per manufacturer’s protocol, Bio-Rad Laboratories, UK) while PGN matter was quantified using both a colorimetric assay and a modified Periodic Acid Schiff (PAS) assay as described (21).

#### Colorimetric Assay

For quantification of crude PGN present within the particles, dyeing of PGN with Remazol Blue Brilliant (RBB; Sigma) was undertaken (22). Briefly, 10 mg PGN were mixed with 1 mL 0.25 M NaOH containing 0.02M RBB and the resulting suspension rotated for the first 6 h at 37°C and overnight at 4°C. The dyed PGN was then washed in cell culture water until the supernatant ran clear to remove excess un-bound RBB. Lysates and supernatant of particles that were prepared using the dyed PGN were read at 595 nm and PGN concentration determined against standard curves of dyed PGN (0–500 μg/mL) prepared in the supernatant or lysate of ACP/BSA particles to match sample matrices.

#### PAS Assay

Since PPD contains residual bacterial polysaccharides, quantification of PPD incorporation was assessed by the modified PAS assay which relies on the formation of a purple complex whose absorbance is proportional to the amount of polysaccharide in solution. Samples and standards (100 μL) were first incubated with 92 mM sodium periodate in 0.5 M sulfuric acid and then with 2.7% sodium arsenite in 0.68 M HCl at room temperature on a plate shaker (40 min). Following the addition of Schiff’s reagent (Merck KGaA, Germany) and a further 30 min incubation at 37°C, the plate was read at 540 nm. Concentration of polysaccharides was determined against a standard curve using PDD (0–250 μg/mL) and prepared in the lysate of AMCP particles to account for sample matrix.

### Immunohistochemistry and Confocal Microscopy

Anonymized, snap frozen human ileal tissue specimens, containing lymphoid patches, were purchased from a commercial tissue bank (Tissue Solutions, UK) and held for use by the authors under HTA license agreement 12383. Samples were from the normal resection margins of three patients with tumors (Table 1). Tissue sections were cryo-sectioned at 14 μm thickness, collected on SuperFrost^®^ slides (Thermo Scientific, USA) and allowed to air dry for 30 min at room temperature.

**Table 1:** **Details of patients from whom ileal tissue specimens were analyzed**.

Gender	Age	Diagnosis
Female	61	Adenocarcinoma of colon
Male	76	Tumor of ileum, carcinoid
Female	65	Tumor of ileum, carcinoid

Tissue sections were fixed in formaldehyde (Sigma-Aldrich, UK) and blocked (10% v/v goat serum, 2% w/v BSA, 0.3 M Glycine). Sections were then incubated with the primary antibody against human IL-10 (ab34843; Abcam, UK), followed by incubation with a secondary antibody (Alexa Fluor^®^ 568 Goat Anti-rabbit IgG [H + L], Invitrogen, UK) and stained for the nanomineral using 0.01 M calcein (Sigma-Aldrich, UK). Finally, the nuclei were counterstained with Hoescht 33342 (H1399, Invitrogen, UK). Sections were imaged with a Leica DMIRE2 microscope (Leica Microsystems, Germany) at 405, 488, or 568 nm, fitted with diode, Ar/ArKr, and HeNe lasers, using a 63×, 1.2 NA water objective lens. Data were recorded using the Leica Confocal Software (v2.61) and images processed using Imaris version 8.0.0 (Bitplane, UK). Data were collected as 8-bit gray scale images and assigned appropriate colors subsequently.

### Cell Isolation, Stimulation, and Cytokine Analysis

The human blood cells obtained for this study were single leukocyte cones purchased from the National Blood Service (UK), peripheral blood mononuclear cells (PBMC) were isolated using density gradient centrifugation. Following isolation, PBMC populations were sometimes then stored (in the short term) at −80°C until required. Cell stimulation and incubations were carried out at 10^6^ cells/mL in RPMI 1640 Media (Sigma-Aldrich) containing 10% fetal calf serum (FCS) (PAA Laboratories), 100 U/mL penicillin, 100 μg/mL streptomycin, and 2 mmol/L l-glutamine (all Sigma-Aldrich), and incubated at 37°C, with 5% CO_2_. Soluble stimulants were added at concentrations of 5 μg/mL PGN (*S. aureus*, Fluka) and/or 10 μg/mL PPD of tuberculin (Statens Serum Institute #2391, Denmark). Controls included 50 μg/mL BSA in PBS (low endotoxin, Sigma-Aldrich) and 1 μg/mL staphylococcal enterotoxin B (positive T cell assay control; Sigma-Aldrich, data not shown). All particulate stimulations were performed by re-suspending 100 μL of freshly synthesized particles in cell culture media containing 10^6^ cells/mL. For cell incubations above 3 h, cells were washed with RPMI and replenished with fresh media (only) and returned to 37°C for the remaining period of incubation. After incubation, cells were centrifuged at 1,500 rpm for 5 min to pellet cells and the supernatant was removed and stored frozen at −80°C until multi-analyte analysis performed by Myriad RBM (Austin, TX, USA), or ELISA according to manufacturers’ instructions (R&D Systems Europe Ltd., Abingdon, UK).

### Flow Cytometric Assays

For surface marker staining, at the end of the incubation period, cells were centrifuged at 1,500 rpm for 5 min to pellet cells and the supernatant removed. The cell pellet was stained with either anti-PD-L1 FITC, PD-L2 PE, and CD14 PerCP Cy5.5, for costimulatory panels or human leukocyte antigen (HLA)-DR FITC and CD14 PerCP Cy5.5 (all BD Biosciences, UK) according to manufacturers’ instructions for 20–40 min on ice in the dark. Cells were then washed with ice cold PBS 1% BSA, re-suspended in a small volume of PBS containing 1% PFA and placed on ice in the dark until acquisition. Single stain compensation tubes for each stain used, as well as an unstained tube, were also prepared from cell samples at this time in order to compensate for spectral overlap. Cells were filtered immediately prior to acquisition on a Cyan-ADP flow cytometer using Summit V4.3.02 software for acquisition and Summit V4.3 for analysis (Beckman Coulter, UK), acquiring a minimum of 250,000 events per sample.

For flow cytometric analysis of particle internalization, particles were prepared as described with the addition of 1 μL of 10 mg/mL Calcein stock solution (Sigma-Aldrich, UK) added to the phosphate solution prior to mixing for each milliliter of mixed solution. Mixed solutions were protected from light thereon and further incubated with rotation at room temperature for 1 h followed by the normal centrifugation and washing once in tissue culture grade water. Then, 100 μL of fluorescently tagged particles (~1.2 × 10^8^ to 1.6 × 10^8^ particles/mL) were added to 10^6^ cells and incubated for 3 h, before washing and re-suspending the cells in ~200 μL ice cold PBS 1% BSA. Cells were then stained for 20 min on ice in the dark for CD14 PerCP Cy5.5 (BD Biosciences, UK) and CD3 Vio-Green (Miltenyi Biotec, UK).

Proliferation assays were carried out as previously described (23). Prior to stimulation, PBMC were stained at 2 × 10^6^/mL with 0.1 μM CFSE (Sigma-Aldrich) diluted in sterile PBS, for 7 min at 37°C in the dark and then washed three times with RPMI 1640 containing 20% FCS. From then onward cells were protected from light. CFSE-stained PBMC (1 × 10^6^ cells/mL) were transferred into FACS tubes and incubated with relevant stimulants as described. On day 5, cells were washed and stained for the surface markers CD3 PE Cy5 and CD4 PE (BD Biosciences, UK) according to manufacturers’ instructions; 500,000 events were acquired for each sample as described previously.

### Flow Imaging Analysis

For Imagestream analysis of particle internalization, particles were synthesized to incorporate calcein, PPD, and PGN as previously described. A 100 μL of fluorescently tagged particles were added to 10^6^ cells/mL and incubated for 3 h, before washing and re-suspending cells in ice cold PBS at 10^7^ cells in 100 μL. Cells were then stained for 20 min on ice in the dark for the surface marker CD14 PerCP Cy5.5 (BD Biosciences, UK), washed, re-suspended in a small volume of PBS, and filtered before acquisition on an ImagestreamX using INSPIRE V4.1.501.0 software (Amnis-Merck Millipore, USA), acquiring a minimum of 50,000 events per sample. A stained particle control was incubated with cells and acquired in addition to unstained particle/cell negative controls and single surface marker controls required for the generation of a compensation matrix and analysis using IDEAS V6.0 software (Amnis-Merck Millipore, USA).

### Statistical Analysis

Statistical evaluation where multiple comparisons were performed were assessed by one way ANOVA and *post hoc* analysis using Tukey’s honestly significant difference method with significance taken as *P* = < 0.05 (particle uptake, induction of surface receptor expression, cytokine production, and T cell proliferation). Only statistically relevant differences are discussed in the text. Experiments comparing two datasets were performed using paired, Student’s *t*-test. *P* = < 0.05 was considered statistically significant.

## Results

### Size and Loading of Synthetic AMCP

**Figure 1:**
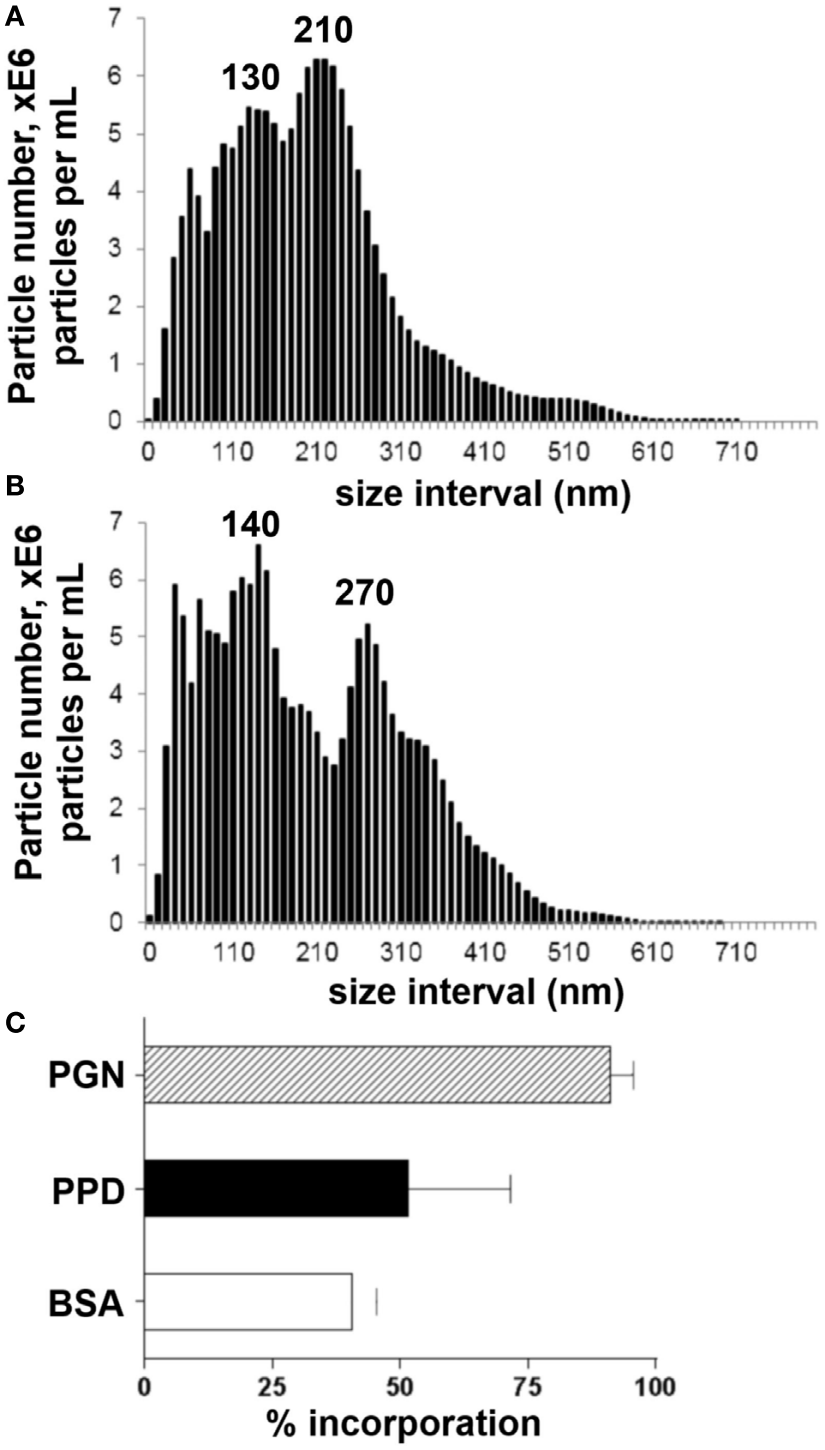
**Synthetic AMCP nanomineral characterization of size and protein incorporation**. **(A)** Size distributions of sAMCP synthesized to incorporate whole protein purified derivative (PPD) antigen and **(B)** sAMCP synthesized to incorporate whole PPD antigen and peptidoglycan (PGN). Data are shown as average of three independent experiments and are displayed as cumulative particle number (E6/mL) per size interval (i.e., every 10 nm). **(C)** Percentage of BSA, PPD, and PGN nanomineral particle incorporation.

To examine the influence of synthetic AMCP (sAMCP) antigen carriage on CD4^+^ antigen-specific T cell responses, sAMCP particles were synthesized and characterized as recently reported (20), but in the presence of PPD of tuberculin from *Mycobacterium tuberculosis*. Resulting particle size distribution, assessed using nanoparticle tracking analysis, revealed a hydrodynamic size (D10–D90) ranging from 67 to 320 nm, a D50 of ~185 nm and peak sizes of about 130 and 210 nm (Figure 1A; Table 2). In some experiments, sAMCP particles were also synthesized to additionally incorporate the bacterial component PGN together with PPD and nanoparticle tracking analysis confirmed that this did not significantly alter the particle size range (i.e., D10–D90: 55–350 nm and D50 of 174 nm, Figure 1B; Table 2, peak sizes of 140 and 270 nm). Thus, overall, sAMCP bearing PPD or PPD together with PGN combined within the same particles demonstrated a similar size range to the naturally occurring endogenous nanomineral (2) (i.e., 75–150 nm) with a small proportion reaching larger agglomerates of ~350 nm, as recently detailed for sAMCP with ovalbumin incorporation (20). The extent of incorporation of PPD antigen was quantified using the PAS assay while trapping of RBB-dyed PGN was assessed directly using colorimetry. With this protocol, sAMCP incorporated around 50% of the total PPD and 90% of the total PGN added during synthesis (Figure 1C). Taken together these data demonstrated high particle loading of our reporter protein antigen (PPD) while maintaining intestinal particle characteristics and size range. In subsequent stimulation assays, particle concentrations were adjusted such that PBMC were exposed to final concentration of ~10 μg/mL PPD and PGN.

**Table 2:** **Descriptive statistics of the synthetic AMCP particle size distributions measured by nanoparticle tracking analysis**.

	D10 (nm; *n* = 3 ± SD)	D50 (nm; *n* = 3 ± SD)	D90 (nm; *n* = 3 ± SD)	Average (nm; *n* = 3 ± SD)
sAMCP/PPD	67 ± 7	184 ± 7	321 ± 38	181 ± 9
sAMCP/PPD/PGN	55 ± 5	174 ± 1	349 ± 28	193 ± 8

*sAMCP/PPD: synthetic amorphous calcium phosphate with a model protein (bovine serum albumin) and recall antigen protein purified derivative (PPD) incorporated*.

*sAMCP/PPD/PGN: synthetic AMCP/PPD with the further incorporation of peptidoglycan*.

*D10, D50, and D90 are sizes calculated with linear interpolation using the two data points above and below the 10th, 50th, and 90th percentile, respectively*.

*See Section “Materials and Methods” for detailed synthesis*.

### Delivery of Protein Antigen in Synthetic AMCP Reduces CD4^+^ T Cell Responses

*In vivo*, endogenously formed AMCP nanoparticles deliver luminal antigen to gut tissue APCs (2). We first sought to examine whether AMCP delivery of antigen influences antigen-specific T cell responses, compared to APC uptake of the same antigen in soluble form. For this we adapted methods described by Singh and Booth, using the MHC class II and costimulation dependant CD4^+^ T cell recall antigen PPD to examine antigen-specific T cell responses (24). In brief, interferon-gamma (IFN-γ) production and proliferation of CD4^+^ T cells within PBMC of PPD-responding donors was measured at 5 days of culture, after a 3 h stimulation of the PBMC with soluble or sAMCP-incorporated PPD.

**Figure 2:**
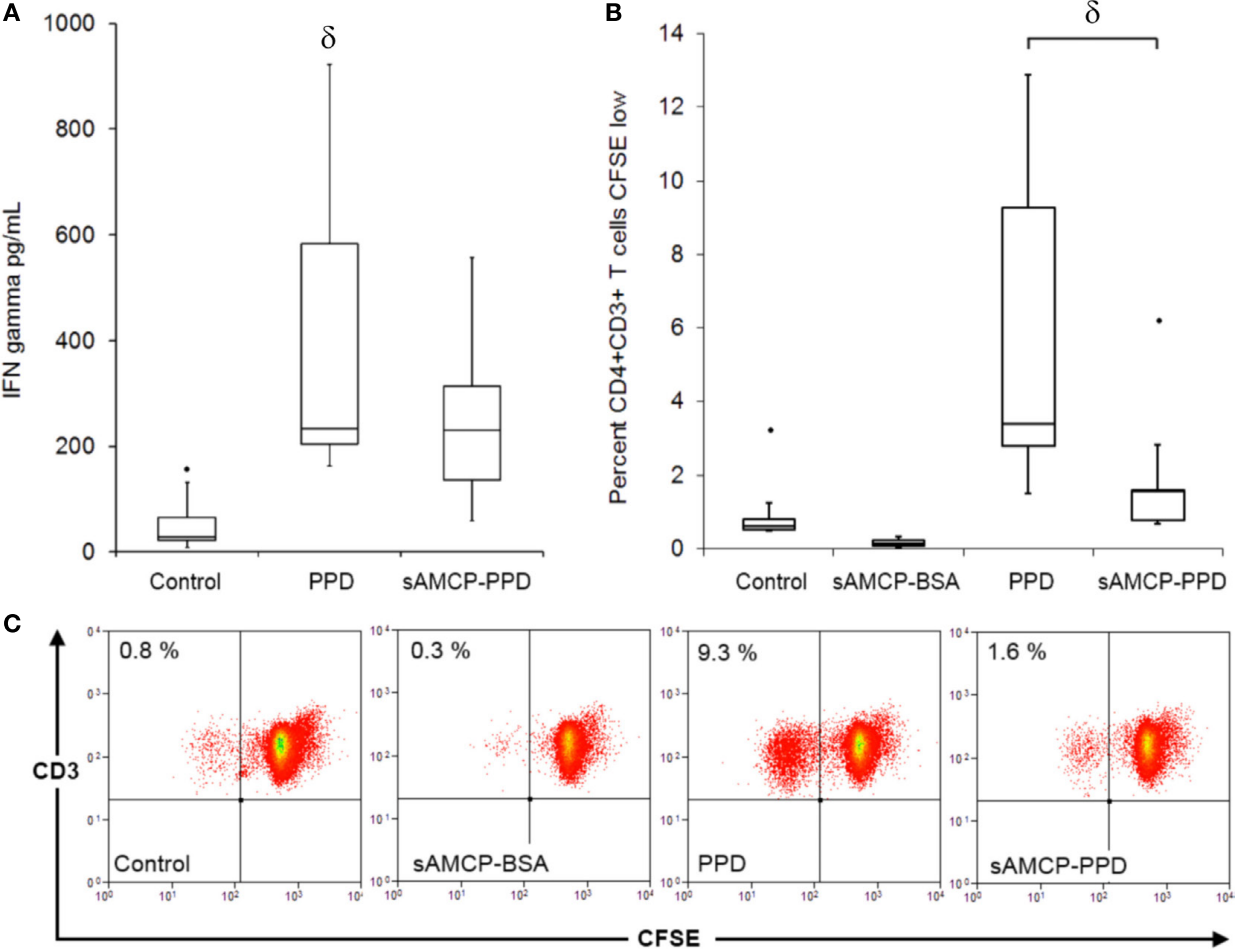
**Antigen-specific CD4^+^ T cell proliferation and interferon (IFN) gamma secretion in response to soluble and sAMCP antigen carriage**. **(A)** Boxplots displaying IFN gamma present in supernatants from peripheral blood mononuclear cells (PBMC) at day 5 in response to protein purified derivative (PPD) and nanomineral carried PPD (sAMCP–PPD) [*n* = 5 PPD responder donors, δ indicates significant difference from the control (assessed by one way ANOVA and *post hoc* analysis using Tukey’s honestly significant difference method, significance taken as *P* = < 0.05)]. Boxplot whiskers were set at 1.5 × interquartile range (IQR) above the third quartile and 1.5 × IQR below the first quartile, minimum or maximum values that have fallen outside this range are shown as outliers (small black dots). **(B)** Boxplots displaying the percentage proliferation of CD4^+^CD3^+^ T cells in PBMC at day 5 in response to soluble PPD (PPD) and nanomineral carried PPD (sAMCP–PPD) (*n* = 5 PPD responder donors, δ *P* = 0.02 PPD versus sAMCP–PPD in a paired Student’s *t*-test). **(C)** Example flow cytometric plots showing CD4^+^CD3^+^ dividing cells (CFSE low) in a CFSE proliferation assay; cells within the lymphocyte gate were gated for CD4 and plotted as CD3 versus CFSE.

Synthetic AMCP carriage of PPD, failed to induce either robust secretion of IFN-γ or CD4^+^ T cell proliferation in PBMC of PPD-responding donors, despite matched loading of nanominerals with the soluble PPD dose. By contrast, exposure to soluble PPD induced both a strong IFN-γ response and proliferation of CD4^+^ T cells (Figures 2A–C). The reduction of T cell responses, when sAMCP-incorporated antigen was taken up by APCs, was not restricted to PPD as the same result was observed with Tetanus Toxoid (Figure S1 in Supplementary Material). These findings were unexpected as we have previously shown that sAMCP is an effective delivery vehicle for APCs, is inert itself, releases its cargo intracellularly, and does not attenuate responsiveness to PGN at the gene level (20). We therefore investigated the uptake of, and CD4^+^ T cell responsiveness, toward sAMCP particles containing both PPD and PGN.

### Immune Responsiveness to sAMCP Dual Antigen-Peptidoglycan Carriage

**Figure 3:**
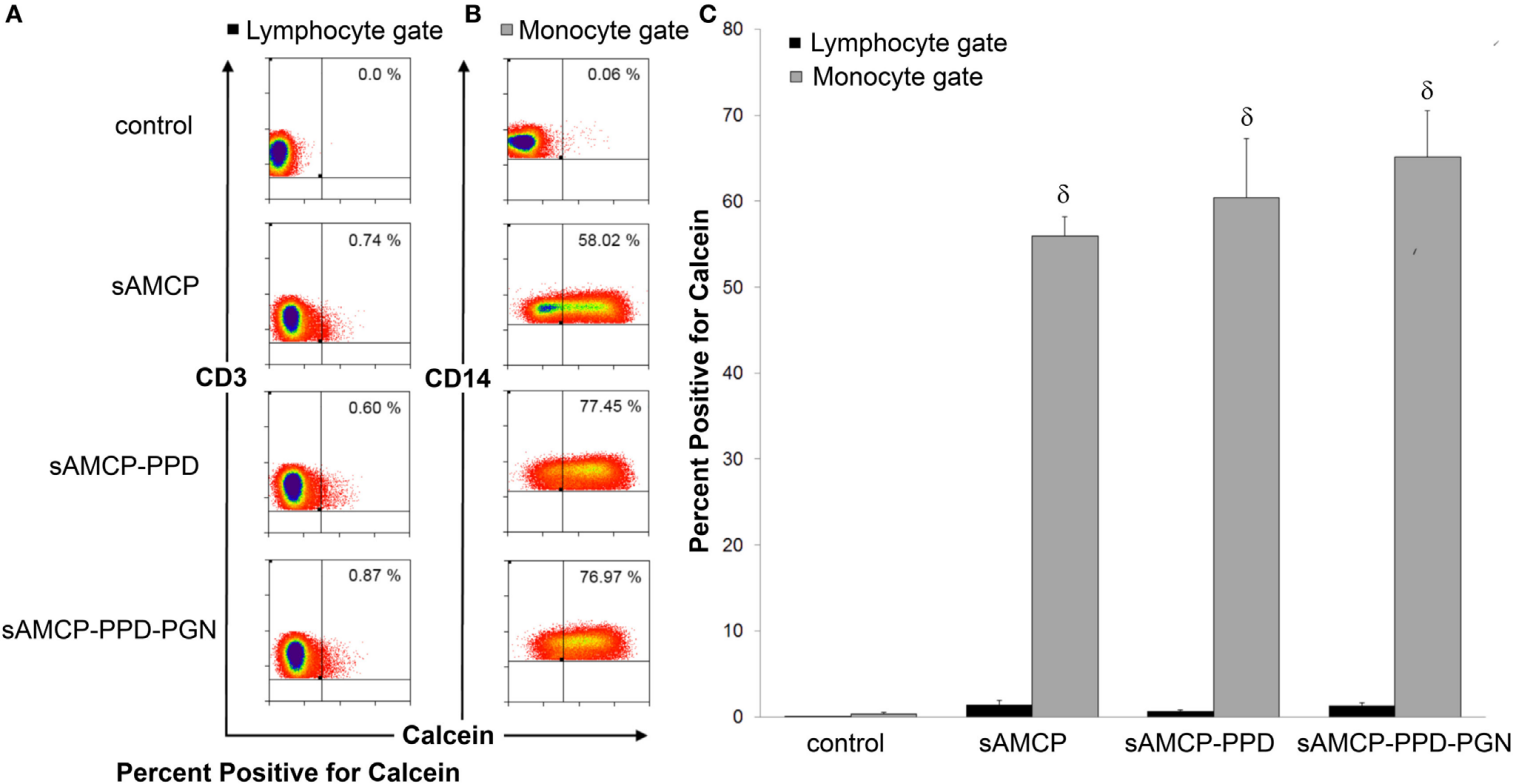
**Synthetic AMCP nanomineral uptake by CD14^+^ cell populations present in peripheral blood mononuclear cells (PBMC)**. Flow cytometric measurement of fluorescently labeled particles in association with **(A)** lymphocyte and **(B)** monocyte gated populations plotted as either CD3 or CD14 versus calcein (full gating strategy is shown in Figure S2 in Supplementary Material). Representative flow plots from one subject are shown in response to incubation with particles containing BSA, PPD, or PPD and peptidoglycan (PGN) are shown. **(C)** Bar chart showing percentage of calcein^+^ cells in lymphocyte (CD3^+^, black fill) and monocyte (CD14^+^, gray fill) populations within PBMC derived from five healthy individuals. δ indicates significant difference from the control (assessed by one way ANOVA and *post hoc* analysis using Tukey’s honestly significant difference method, significance taken as *P* = < 0.05).

**Figure 4:**
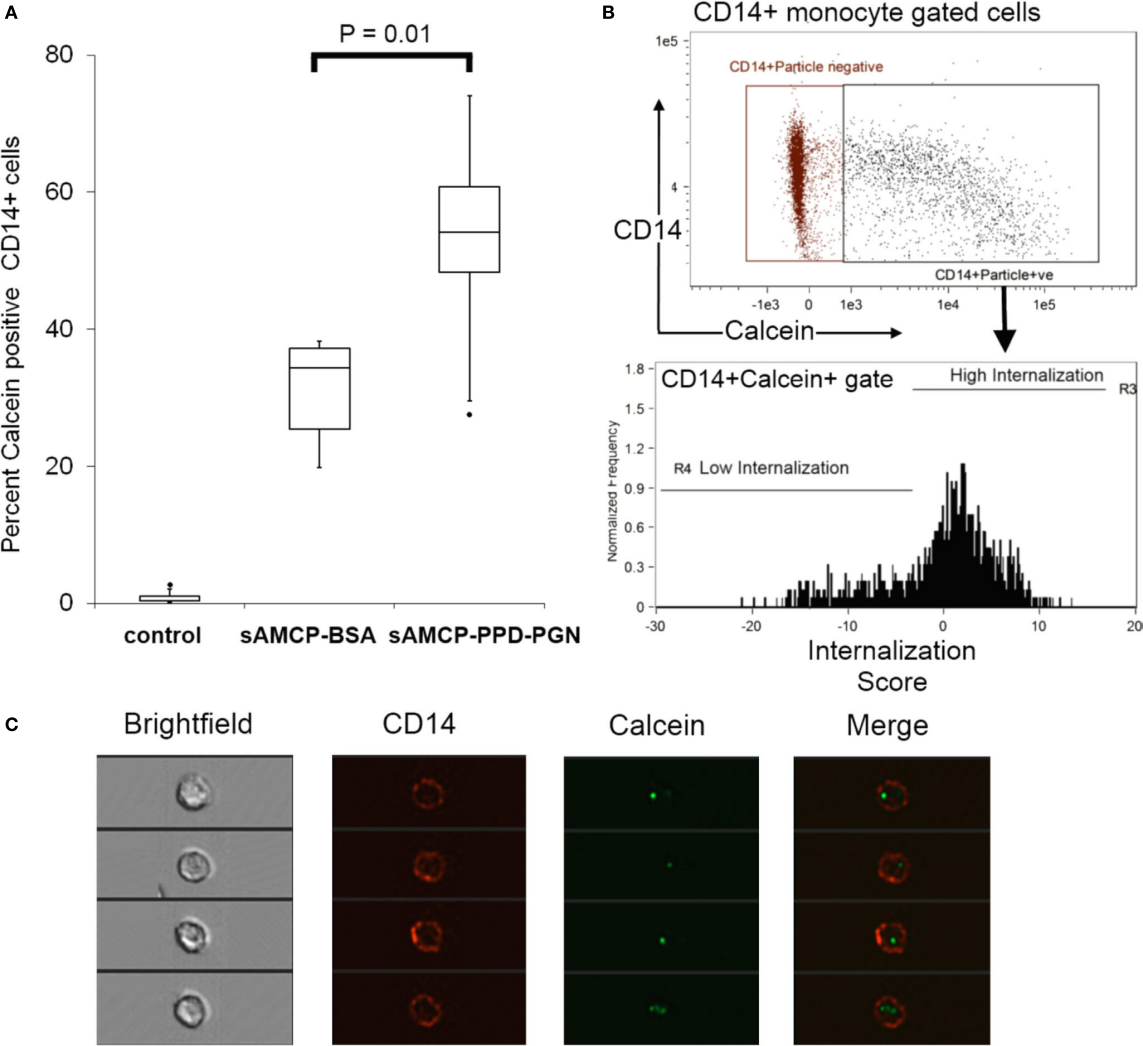
**Synthetic AMCP nanomineral internalization by CD14^+^ cell populations present in peripheral blood mononuclear cells (PBMC)**. ImagestreamX analysis of particle internalization. **(A)** Boxplots showing the percentage of calcein^+^CD14^+^ within PBMC from five healthy donors (*P* = 0.01 sAMCP versus sAMCP–PPD–peptidoglycan (PGN) in a paired Student’s *t*-test). Cells residing in the CD14^+^calcein^+^ double positive gate shown were assessed for the degree of particle internalization (defined as calcein fluorescence residing within the CD14 fluorescence marking the cell wall boundary), cell gates are shown in panel **(B)**, full gating strategy is shown in Figure S3 in Supplementary Material. **(C)** Example images of CD14^+^calcein^+^ cells residing within the high internalization gate are shown. For boxplots, whiskers were set at 1.5 × interquartile range (IQR) above the third quartile and 1.5 × IQR below the first quartile, minimum or maximum values that have fallen outside this range are shown as outliers (small black dots).

First, we examined the efficiency of uptake of PPD + PGN-loaded sAMCP by APCs present in PBMC. Synthetic AMCP particles were synthesized as described in the previous sections, but in the presence of the fluorescent marker calcein (20), and loaded with PPD and/or PGN. Control sAMCP particles were loaded with BSA only. All particles were incubated with PBMC for 3 h. Using flow cytometry and flow imaging techniques internalization of sAMCP was assessed in both CD3^+^ T lymphocytes and CD14^+^ monocytes. Calcein^+^ particle staining, representing AMCP uptake, corresponded almost exclusively with cells of the monocytic lineage. Almost 60% of CD14^+^ monocyte/macrophage cells were positive for calcein after 3 h sAMCP exposure, compared to less than 1% of the CD3^+^ lymphocyte population (Figure 3). Flow imaging confirmed that sAMCP–PPD–PGN particles were taken up more readily than sAMCP control particles (sAMCP–BSA), and that the particles were not just associated with monocytes but were internalized, with 51.1 ± 10.5% SEM defined as highly internalized, visualized residing well within the CD14-defined cell membrane boundary (Figure 4).

**Figure 5:**
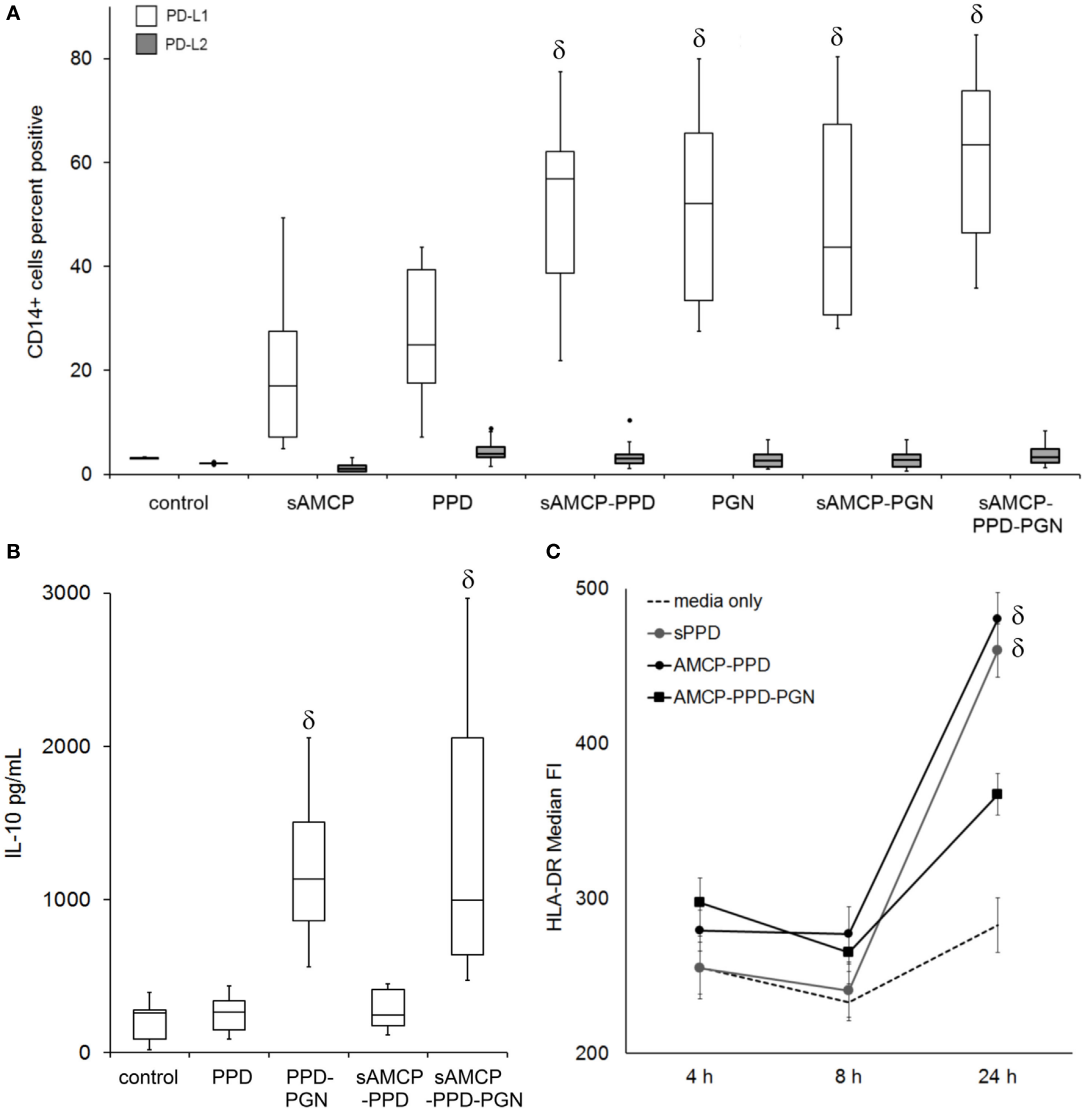
**The influence of synthetic AMCP on programmed death receptor ligand 1 (PD-L1), PD-L2, IL-10 secretion, and MHC class II expression**. **(A)** Changes in the surface expression of PD-L1 (no fill) and PD-L2 (gray fill) on CD14^+^ gated monocytic cells present within peripheral blood mononuclear cells (PBMC), data from six donors CD14^+^ cell responses to all stimulants tested are shown (example histogram plots from one donor are shown in Figure S4 in Supplementary Material). **(B)** IL-10 present in supernatants of PBMC day 5 after stimulation, averaged data from six donors are shown. **(C)** Median fluorescence intensity of HLA-DR surface expression over time after PPD and nanomineral PPD (sAMCP–PPD) or nanomineral PPD/peptidoglycan (PGN) (sAMCP–PPD–PGN) stimulation, averaged data (±SEM) from five donors are shown. δ indicates significant difference from the control (assessed by one way ANOVA and *post hoc* analysis using Tukey’s honestly significant difference method, significance taken as *P* = < 0.05). For boxplots, whiskers were set at 1.5 × interquartile range (IQR) above the third quartile and 1.5 × IQR below the first quartile, minimum or maximum values that have fallen outside this range are shown as outliers (small black dots).

As well as being pro-inflammatory, under certain circumstances PGN is known to play important roles in countering inflammatory responses and inducing tolerogenic responses through the secretion of IL-10 and the expression of PD-L1 by APCs (10, 13, 25–28). Hence, we next studied the effects of sAMCP-delivered PPD/PGN on primary PBMC cultures in terms of (i) the induction of the cell surface regulatory coreceptors PD-L1 and PD-L2, (ii) IL-10 secretion, and (iii) modulation of the HLA which is associated with MHC class II presentation of exogenously derived antigen. The delivery of PPD in AMCP form (but not in suspension) increased PD-L1 expression by CD14^+^ APCs. As anticipated (and in agreement with previous studies where PGN was present in suspension), the addition of PGN also stimulated the expression of PD-L1, but not PD-L2, by the APCs and additionally stimulated IL-10 secretion (Figures 5A,B). Furthermore, cell surface expression of the MHC class II antigen presenting molecule, HLA-DR, was significantly increased on APCs in response to PPD by 24 h only when PGN was absent (Figure 5C).

**Figure 6:**
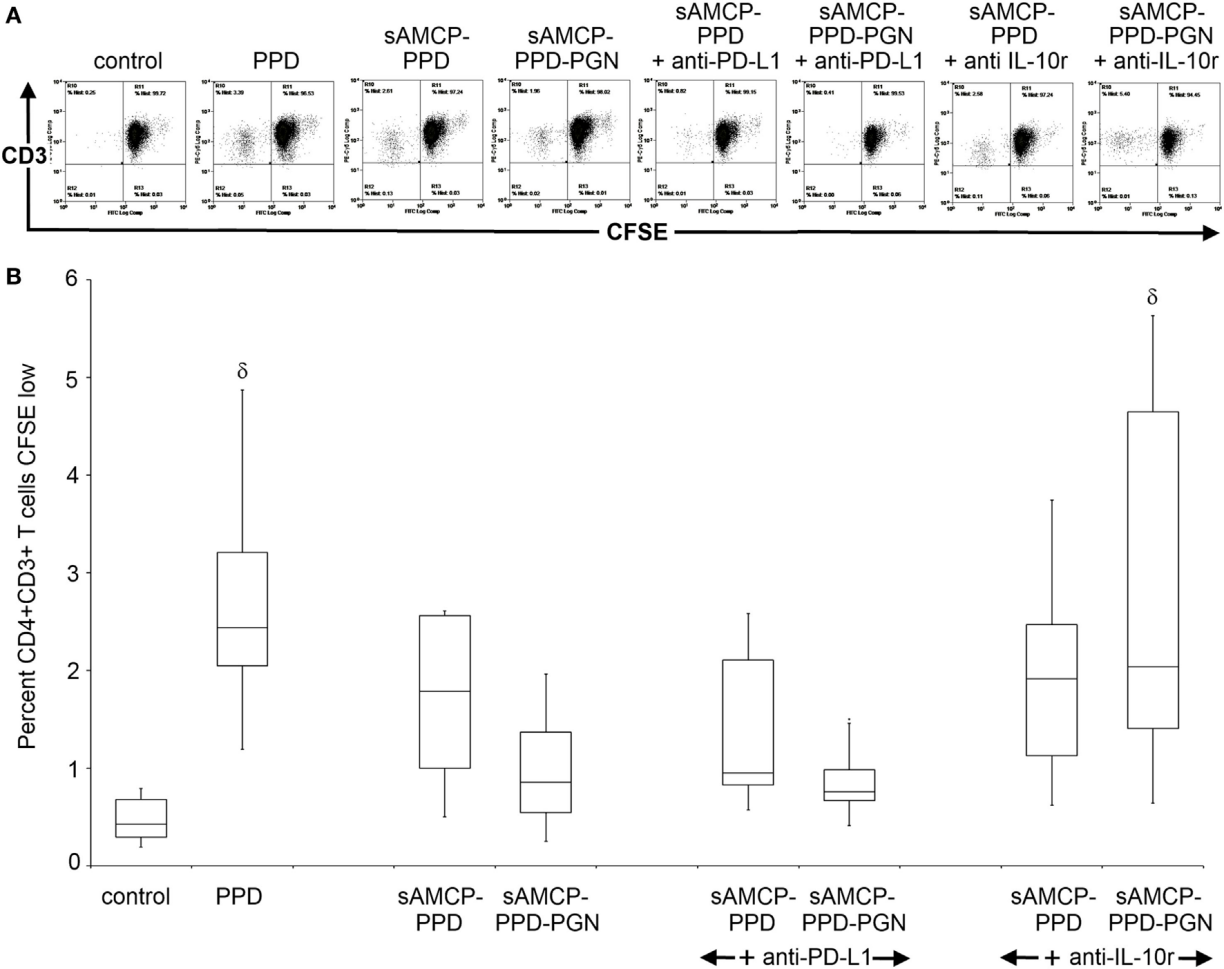
**Antigen-specific CD4^+^ T cell proliferation in response to sAMCP codelivery of antigen and peptidoglycan (PGN)**. **(A)** Example flow cytometric plots of CD4^+^CD3^+^ gated T cells within peripheral blood mononuclear cells (PBMC) showing CFSE dilution. For analysis, an extended lymphocyte gate was drawn based on the forward and side scatter profiles of lymphocytes within PBMC, followed by a CD4^+^ gate and subsequent CD3 gate. CD3^+^CD4^+^ cells were then finally plotted as CD3 versus CFSE and a quadrant drawn based on the negative control to measure the percentage of dividing (CFSE low) cells in response to antigen-specific stimulation. Example analysis plots of responses from one donor are shown. **(B)** Boxplots displaying percentage of divided CD4^+^CD3^+^ T cells at day 5 after stimulation with PPD, nanomineral PPD (sAMCP–PPD), or nanomineral PPD/PGN (sAMCP–PPD–PGN), with or without blocking antibodies for programmed death receptor ligand 1 (PD-L1) or the IL-10 receptor (*n* = 6 PPD responders). δ indicates significant difference from the control (assessed by one way ANOVA and *post hoc* analysis using Tukey’s honestly significant difference method, significance taken as *P* = < 0.05). For boxplots, whiskers were set at 1.5 × interquartile range (IQR) above the third quartile and 1.5 × IQR below the first quartile, minimum or maximum values that have fallen outside this range are shown as outliers (small black dots).

We therefore considered the mechanism by which intracellular delivery to APCs of PGN plus PPD antigen could influence the proliferation of antigen-responding T cells within PBMC derived from a further six healthy, PPD-responding, donors. Antigen-specific CD4^+^ T cell proliferation assays were performed as before (Figure 2) but with the addition of blocking antibodies against either PD-L1 or the IL-10 receptor (IL-10r). Consistent with our previous results, delivery of PPD in sAMCP reduced CD4^+^ T cell proliferation, and this inhibition was further enhanced when PGN was present (Figure 6). Blocking the IL-10r and hence inhibiting IL-10 activity restored T cell proliferation, whereas the neutralization of PD-L1 had no such effect (Figure 6). Taken together our data show that multiple checkpoints appear to inhibit T cell proliferation in response to sAMCP-delivered antigen. First, the nanomineral itself does not permit efficient protein presentation for T cell proliferation in comparison to delivery of free protein. Second, the concomitant carriage of PGN, which occurs *in vivo*, reduces the available HLA-DR on the surface of the APC thereby limiting antigen presentation *via* the effect of IL-10 production. Finally, antigen that is presented occurs in the context of immuno-inhibitory PD-L1 (Figure 5).

**Figure 7:**
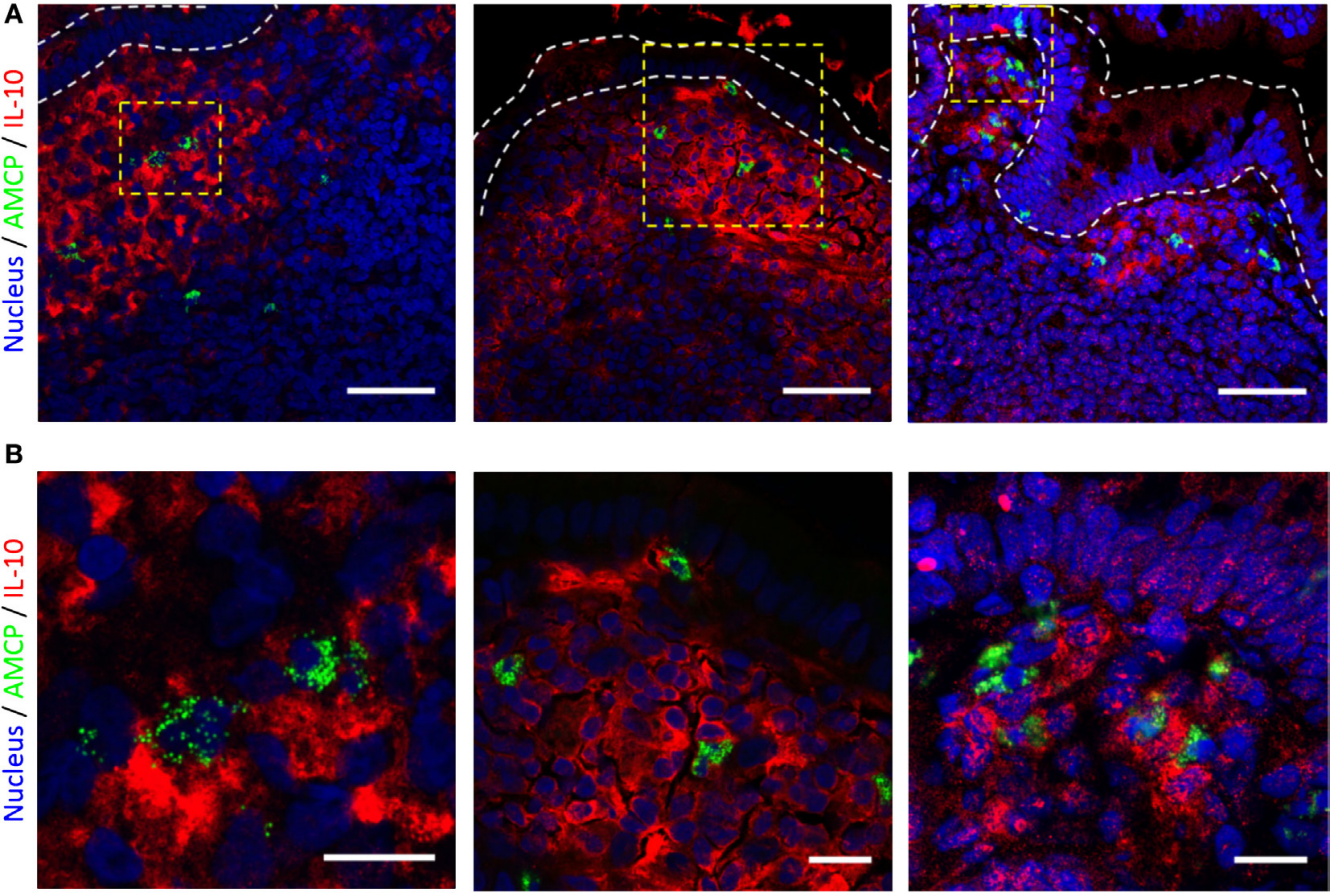
**IL-10 and amorphous magnesium-substituted calcium phosphate (AMCP) staining of human ileal lymphoid patches**. **(A)** 63× confocal micrographs from tissue sections from three individuals. IL-10 staining is shown in red and AMCP nanomineral staining in green. Staining is localized to cells residing with the sub-epithelial dome of ileal lymphoid patches, nuclear staining is shown in blue. Scale bars—50 μm. **(B)** Increased magnifications of area’s bounded by yellow boxes within the micrographs shown.

These studies indicated that the secretion of IL-10 provided the most striking attenuation of antigen-specific CD4 T cell proliferation in response to nanomineral carried PPD–PGN. To verify the physiological relevance of these observations, we therefore sought to confirm that AMCP nanomineral-bearing cells in the Peyer’s patch sub-epithelial dome (SED) exist in an IL-10 high environment. Samples of human tissue, from three patients, containing Peyer’s patches were collected from the uninvolved margins of surgically resected intestinal specimens. AMCP^+^ cells of the SED region were detected by calcein staining and IL-10 protein was detected by specific antibody-based staining. Consistent with previous reports, all specimens showed clear calcein staining in the SED representing the presence of AMCP (29). In addition, IL-10 staining was strongly positive in the same area compared to the rest of the patch (Figure 7). Thus, our findings imply that the failure to induce antigen-specific CD4 T cell proliferation, observed *in vitro* in response to antigen and PGN codelivered to APCs within sAMCP nanomineral, is of physiological relevance *in vivo* as the key attenuating molecule (IL-10) appears abundantly present in close proximity to AMCP.

## Discussion

Antigen-presenting cell interactions with microbiota are of substantial importance in maintaining the balance between tolerance and immunity in the gut (14–18). The discovery of endogenous intestinal nanominerals offers an additional route by which luminal antigen and components of intestinal microbiota may be continually sampled. Nanomineral carriers of luminal components are well placed to play a role in the microbiota—immune system dialog (2). Using an *in vitro* PBMC-based framework we have examined the influence of an intestinal nanomineral mimetic in a manner that will inform future *in vivo* studies. Consistent with other reports of uptake of nano-sized materials (30, 31), the nanomineral mimetic was rapidly sequestered by professional phagocytes within PBMC irrespective of cargo. We previously hypothesized that the nanomineral pathway might ensure tolerogenic signaling in association with harmless luminal derived antigen, by facilitating the codelivery of antigen with mucosal bacterial components such as PGN (2). In the unique environment of the gut, this might ensure that adaptive immune responses to “harmless antigen” are not overly dominant, while retaining the opportunity for protective immune responses if the same antigen were identified in a different context (i.e., without codelivery). We now show that synthetic mimetics of intestinal nanomineral carriers loaded with antigen and PGN are *themselves* capable of attenuating CD4^+^ T cell responses, resulting in reduced T cell proliferation to a cognate recall antigen, and do not simply rely upon a hypo-responsive environment (such as the gut) for this effect. We demonstrated the PGN promotion of blood derived monocytes into a phenotype less suited to the promotion of Th1 type CD4^+^ T cell responses, augmented by the dual delivery of antigen and PGN to APCs by sAMCP, chiefly regulated by the secretion of IL-10, a classically anti-inflammatory cytokine. Several murine studies have reported the occurrence of IL-10 secreting APC populations in the maintenance of intestinal tolerance, especially pertaining to the lamina propria and Peyer’s patches (32–38). Although human studies are considerably more limited, IL-10 production has also been identified by ELISPOT and RT-PCR analysis of mononuclear cells freshly isolated from human Peyer’s patch biopsies (39). *In vivo*, it is possible that IL-10 production may be exclusive to AMCP recipient APCs or additionally sustained by the subsequent generation or stimulation of regulatory T cells. Early exposure to TLR agonists has previously been demonstrated *in vitro* to block the differentiation of monocytes into mature APC, resulting in a PD-L1^+^ tolerogenic APC phenotype, which fail to induce Th1 cell proliferation, instead inducing regulatory T cells (10). Tolerogenic, regulatory T cell inducing macrophage subsets have similarly been reported to reside in both the lamina propria and Peyer’s patches of mice (34–36, 38). Further work should therefore consider the role of AMCP on the influence of T cell responsiveness and the generation of IL-10 and regulatory T cells *in vivo*, both of which could drive a tolerogenic response.

Parallels exist between naturally forming intestinal nanominerals and nanoparticulate calcium phosphate vaccine preparations. Both possess distinctly adventitious carriage and targeted delivery of antigen simultaneously with additional immune modulators to cells with professional antigen-presenting ability. Calcium phosphate nanoparticulate species are generally understood to adsorb biological materials well and have been used as adjuvants reported to enhance immune responses (40, 41). As naturally occurring compounds, calcium phosphate species are considered to be well tolerated with few side effects and have additionally been utilized as vectors for gene delivery (42). We found that loading sAMCP with PPD antigen alone (i.e., without PGN) led to significantly reduced CD4 T cell responses. A possible explanation for the failure of T cell responsiveness to sAMCP-delivered antigen in the work reported here (i.e., Figure 2) is that the PRR ligand PGN was lacking. Seminal work by Blander and Medzhitov examining phagosome maturation has described the existence of discrete pathways for handling exogenously derived particulate antigen of differing origin at the subcellular level. Phagosome-autonomous control of antigen presentation dictates that phagosomes containing particulate cargo that engages TLRs are favored for MHC class II presentation. Whereas non-pathogen associated particulate material (i.e., devoid of PRR ligands), such as apoptotic cells, is destined to follow distinct endosomal pathways, protecting the host from the inappropriate presentation of self-antigen (9, 43, 44). Thus, phagocytosed sAMCP containing the purified protein antigen PPD, in the absence of PGN, may be more susceptible to complete phagosomal degradation rather than continuation along the MHC class II presentation pathway. Compartmentalized intracellular pathways for phagocytised material are not unique to the uptake of apoptotic self and may also have a role in the processing and presentation of exogenous particulate antigen. Several groups have reported same particle (same phagosome) delivery of antigen and immune stimulant (PRR ligands) are required to elicit robust T cell responses to exogenous particulate antigen (40, 45–47). Additional to compartmentalized pathways for phagocytosed material, it is also likely that whole protein antigen taken up by APCs in nanoparticulate form may result in modified processing and presentation of the antigenic protein cargo itself, compared to the same protein alone or in its native form. Degradation of the sAMCP–PPD protein construct may result in protein epitopes that may be either inefficiently processed or conversely destroyed during the processing steps within APCs. Variations occurring at the MHC II loading compartments may result in the presentation of sub-optimal or cryptic peptides (48). Recent studies suggest that variations in MHC processing and presentation pathways apply to innocuous nanomaterials and that exploitation of these pathways is possible for therapeutic benefit (49, 50). While such studies support our observations, further investigations are required to fully delineate the cellular handling of sAMCP, the processing and presentation of its cargo and the influence of both PRR and NOD ligands on this process, in health and disease.

Our data show that loading synthetic AMCP with T cell antigen in combination with bacterial PGN also fails to potentiate immune responses to CD4^+^ T cell antigen. Instead, through the modulation of cell surface MHC expression and IL-10 secretion the sAMCP–antigen–PGN construct markedly reduces antigen-specific CD4^+^ T cell responses. MHC class II molecules are constitutively expressed by professional APC, but expression is further modulated by multiple agents associated with immune responses. The downregulation of cell surface MHC II molecules is understood to be one mechanism by which IL-10 suppresses adaptive immune responses (51, 52). In addition, the instruction of cell surface PD-L1 expression might also determine the type of T cell that proliferates, and may favor regulatory over inflammatory T cells (26, 27). In the intestine, recognition of resident microbiota *via* PRRs appears a seminal feature in the maintenance of tolerance, as well as the development of inflammatory disease in susceptible individuals (14–18). The intestine lymphoid patches and mesenteric lymph nodes are both sites where AMCP^+^ cells are observed *in vivo*—presumably arriving at the latter *via* the former. This work demonstrates the importance of IL-10 in determining the extent of T cell immune responsiveness to luminal derived antigen delivered *via* nanominerals under steady state conditions. Recognition and cellular handling of bacterial components carried by AMCP intestinal nanomineral appear an important part of the apparent immuno-regulatory feature of intestinal nanominerals. Defects in the bacterial sensing receptor NOD-2 and polymorphisms in genes associated with the intracellular handling of bacteria, or their components for processing and MHC class II presentation are associated with the development of CD. Interruption of these processes may heavily influence or even disrupt the intestinal nanomineral pathway (19). Defective recognition of the microbial component PGN *via* the NOD-2 moiety muramyl dipeptide results in a failure to upregulate the immuno-inhibitory receptor PD-L1, observed *in vitro* and *in situ* in CD intestinal lymphoid tissue, importantly, exhibited specifically by nanomineral-bearing APCs residing in Peyer’s Patches (25, 29). Our findings further exemplify the inert, silent nature of AMCP intestinal nanominerals, which appear to act as chaperones for the codelivery of protein antigen and bacterial components in the context of T helper cell responses. Increasing research strategies exploring the use of nanoparticle based delivery systems designed for immunotherapeutic benefit, such as allergen immunotherapy (53, 54) together with this current study, continue to demonstrate the broad potential for the application of nanoparticulate dual delivery systems.

## Author Contributions

JP and RH together developed the overall hypothesis, designed the study, and wrote the manuscript. RH and LP undertook particle synthesis and particle sizing analysis. LP was responsible for the quantification of organic matter within AMCP particles and together with CH carried out cytokine analysis. RH performed cell isolation, stimulation, flow cytometric, and flow imaging analyses. JR was responsible for immunohistochemistry and confocal analysis. All authors contributed to data interpretation and to the critical review of the manuscript.

## Conflict of Interest Statement

The authors have no conflict of interests but note that the MRC have filed a patent for the synthetic AMCP material and its potential therapeutic applications, and some of the authors are attributed as inventors and/or contributors.

## References

[1] Lomer MC Thompson RP Powell JJ. Fine and ultrafine particles of the diet: influence on the mucosal immune response and association with Crohn’s disease. Proc Nutr Soc (2002) 61(1):123–30. 10.1079/PNS2001134 12002786

[2] Powell JJ Thomas-McKay E Thoree V Robertson J Hewitt RE Skepper J An endogenous nanomineral chaperones luminal antigen and peptidoglycan to intestinal immune cells. Nat Nanotechnol (2015) 10:361–9. 10.1038/nnano.2015.19 25751305 PMC4404757

[3] Takeuchi O Akira S Pattern recognition receptors and inflammation. Cell (2010) 140(6):805–20. 10.1016/j.cell.2010.01.022 20303872

[4] Smith DM Simon JK Baker JR Jr. Applications of nanotechnology for immunology. Nat Rev Immunol (2013) 13(8):592–605. 10.1038/nri3488 23883969 PMC7097370

[5] Slütter B Plapied L Fievez V Sande MA des Rieux A Schneider YJ Mechanistic study of the adjuvant effect of biodegradable nanoparticles in mucosal vaccination. J Control Release (2009) 138(2):113–21. 10.1016/j.jconrel.2009.05.011 19445980

[6] De Temmerman ML Rejman J Demeester J Irvine DJ Gander B De Smedt SC. Particulate vaccines: on the quest for optimal delivery and immune response. Drug Discov Today (2011) 16(13–14):569–82. 10.1016/j.drudis.2011.04.006 21570475

[7] Commins SP Borish L Steinke JW. Immunologic messenger molecules: cytokines, interferons, and chemokines. J Allergy Clin Immunol (2010) 125(2 Suppl 2):S53–72. 10.1016/j.jaci.2009.07.008 19932918

[8] Wang S Chen L. T lymphocyte co-signaling pathways of the B7-CD28 family. Cell Mol Immunol (2004) 1(1):37–42. 16212919

[9] Blander JM Medzhitov R. Toll-dependent selection of microbial antigens for presentation by dendritic cells. Nature (2006) 440(7085):808–12. 10.1038/nature04596 16489357

[10] Wölfle SJ Strebovsky J Bartz H Sähr A Arnold C Kaiser C PD-L1 expression on tolerogenic APCs is controlled by STAT-3. Eur J Immunol (2011) 41(2):413–24. 10.1002/eji.201040979 21268011

[11] Nahid MA Yao B Dominguez-Gutierrez PR Kesavalu L Satoh M Chan EK. Regulation of TLR2-mediated tolerance and cross-tolerance through IRAK4 modulation by miR-132 and miR-212. J Immunol (2013) 190(3):1250–63. 10.4049/jimmunol.1103060 23264652 PMC3552145

[12] Hedl M Abraham C. Secretory mediators regulate Nod2-induced tolerance in human macrophages. Gastroenterology (2011) 140(1):231–41. 10.1053/j.gastro.2010.09.009 20854823 PMC3145247

[13] Samarasinghe R Tailor P Tamura T Kaisho T Akira S Ozato K. Induction of an anti-inflammatory cytokine, IL-10, in dendritic cells after toll-like receptor signaling. J Interferon Cytokine Res (2006) 26(12):893–900. 10.1089/jir.2006.26.893 17238832

[14] Rakoff-Nahoum S Paglino J Eslami-Varzaneh F Edberg S Medzhitov R. Recognition of commensal microflora by toll-like receptors is required for intestinal homeostasis. Cell (2004) 118(2):229–41. 10.1016/j.cell.2004.07.002 15260992

[15] Kubinak JL Round JL Toll-like receptors promote mutually beneficial commensal-host interactions. PLoS Pathog (2012) 8(7):e1002785 10.1371/journal.ppat.1002785 22910541 PMC3406078

[16] Round JL O’Connell RM Mazmanian SK. Coordination of tolerogenic immune responses by the commensal microbiota. J Autoimmun (2010) 34(3):J220–5. 10.1016/j.jaut.2009.11.007 19963349 PMC3155383

[17] Feng T Elson CO. Adaptive immunity in the host-microbiota dialog. Mucosal Immunol (2011) 4(1):15–21. 10.1038/mi.2010.60 20944557 PMC4557730

[18] Kamada N Núñez G. Regulation of the immune system by the resident intestinal bacteria. Gastroenterology (2014) 146(6):1477–88. 10.1053/j.gastro.2014.01.060 24503128 PMC3995843

[19] Xavier RJ Podolsky DK. Unravelling the pathogenesis of inflammatory bowel disease. Nature (2007) 448(7152):427–34. 10.1038/nature06005 17653185

[20] Pele LC Haas CT Hewitt RE Robertson J Skepper J Brown A Synthetic mimetics of the endogenous gastrointestinal nanomineral: silent constructs that trap macromolecules for intracellular delivery. Nanomedicine (2016) 13:619–30. 10.1016/j.nano.2016.07.008 27478107 PMC5339085

[21] Jugdaohsingh R Soluble Silica and Aluminium Bioavailability. Ph.D. thesis, University of London, London (1999).

[22] Zhou R Chen S Recsei P A dye release assay for determination of lysostaphin activity. Anal Biochem (1988) 171(1):141–4. 3407910 10.1016/0003-2697(88)90134-0

[23] Lyons AB. Analysing cell division in vivo and in vitro using flow cytometric measurement of CFSE dye dilution. J Immunol Methods (2000) 243(1–2):147–54. 10.1016/S0022-1759(00)00231-3 10986412

[24] Singh SD Booth CG. Tuberculin-induced lymphocyte proliferation in whole blood: an antigen specific method for assessing immunosuppressive agents. J Immunol Methods (2002) 260(1–2):149–56. 10.1016/S0022-1759(01)00557-9 11792385

[25] Hewitt RE Pele LC Tremelling M Metz A Parkes M Powell JJ Immuno-inhibitory PD-L1 can be induced by a peptidoglycan/NOD2 mediated pathway in primary monocytic cells and is deficient in Crohn’s patients with homozygous NOD2 mutations. Clin Immunol (2012) 143(2):162–9. 10.1016/j.clim.2012.01.016 22397822

[26] Fukaya T Takagi H Sato Y Sato K Eizumi K Taya H Crucial roles of B7-H1 and B7-DC expressed on mesenteric lymph node dendritic cells in the generation of antigen-specific CD4^+^Foxp3^+^ regulatory T cells in the establishment of oral tolerance. Blood (2010) 116(13):2266–76. 10.1182/blood-2009-10-250472 20574047 PMC3368550

[27] Francisco LM Salinas VH Brown KE Vanguri VK Freeman GJ Kuchroo VK PD-L1 regulates the development, maintenance, and function of induced regulatory T cells. J Exp Med (2009) 206(13):3015–29. 10.1084/jem.20090847 20008522 PMC2806460

[28] Francisco LM Sage PT Sharpe AH. The PD-1 pathway in tolerance and autoimmunity. Immunol Rev (2010) 236:219–42. 10.1111/j.1600-065X.2010.00923.x 20636820 PMC2919275

[29] Robertson J Haas CT Pele LC Monie TP Charalambos C Parkes M Intestinal APCs of the endogenous nanomineral pathway fail to express PD-L1 in Crohn’s disease. Sci Rep (2016) 6:26747. 10.1038/srep26747 27226337 PMC4880906

[30] Yue H Wei W Yue Z Lv P Wang L Ma G Particle size affects the cellular response in macrophages. Eur J Pharm Sci (2010) 41(5):650–7. 10.1016/j.ejps.2010.09.006 20870022

[31] Uto T Akagi T Toyama M Nishi Y Shima F Akashi M Comparative activity of biodegradable nanoparticles with aluminum adjuvants: antigen uptake by dendritic cells and induction of immune response in mice. Immunol Lett (2011) 140(1–2):36–43. 10.1016/j.imlet.2011.06.002 21693134

[32] Denning TL Wang YC Patel SR Williams IR Pulendran B. Lamina propria macrophages and dendritic cells differentially induce regulatory and interleukin 17-producing T cell responses. Nat Immunol (2007) 8(10):1086–94. 10.1038/ni1511 17873879

[33] Manicassamy S Pulendran B. Modulation of adaptive immunity with toll-like receptors. Semin Immunol (2009) 21(4):185–93. 10.1016/j.smim.2009.05.005 19502082 PMC4125416

[34] Iwasaki A Kelsall BL. Unique functions of CD11b^+^, CD8 alpha^+^, and double-negative Peyer’s patch dendritic cells. J Immunol (2001) 166(8):4884–90. 10.4049/jimmunol.166.8.4884 11290765

[35] Takada Y Hisamatsu T Kamada N Kitazume MT Honda H Oshima Y Monocyte chemoattractant protein-1 contributes to gut homeostasis and intestinal inflammation by composition of IL-10-producing regulatory macrophage subset. J Immunol (2010) 184(5):2671–6. 10.4049/jimmunol.0804012 20107182

[36] Tsuji NM Mizumachi K Kurisaki J. Interleukin-10-secreting Peyer’s patch cells are responsible for active suppression in low-dose oral tolerance. Immunology (2001) 103(4):458–64. 10.1046/j.1365-2567.2001.01265.x 11529936 PMC1783258

[37] Monteleone I Platt AM Jaensson E Agace WW Mowat AM IL-10-dependent partial refractoriness to toll-like receptor stimulation modulates gut mucosal dendritic cell function. Eur J Immunol (2008) 38(6):1533–47. 10.1002/eji.200737909 18461564 PMC2988418

[38] Tsuji NM Mizumachi K Kurisaki J. Antigen-specific, CD4+CD25+ regulatory T cell clones induced in Peyer’s patches. Int Immunol (2003) 15(4):525–34. 10.1093/intimm/dxg051 12663682

[39] Hauer AC Bajaj-Elliott M Williams CB Walker-Smith JA MacDonald TT. An analysis of interferon gamma, IL-4, IL-5 and IL-10 production by ELISPOT and quantitative reverse transcriptase-PCR in human Peyer’s patches. Cytokine (1998) 10(8):627–34. 10.1006/cyto.1997.0337 9722936

[40] Heit A Schmitz F Haas T Busch DH Wagner H. Antigen co-encapsulated with adjuvants efficiently drive protective T cell immunity. Eur J Immunol (2007) 37(8):2063–74. 10.1002/eji.200737169 17628858

[41] Relyveld EH. Preparation and use of calcium phosphate adsorbed vaccines. Dev Biol Stand (1986) 65:131–6. 3549396

[42] Giger EV Puigmartí-Luis J Schlatter R Castagner B Dittrich PS Leroux JC. Gene delivery with bisphosphonate-stabilized calcium phosphate nanoparticles. J Control Release (2011) 150(1):87–93. 10.1016/j.jconrel.2010.11.012 21111013

[43] Blander JM Medzhitov R. Regulation of phagosome maturation by signals from toll-like receptors. Science (2004) 304(5673):1014–8. 10.1126/science.1096158 15143282

[44] Blander JM Medzhitov R. On regulation of phagosome maturation and antigen presentation. Nat Immunol (2006) 7(10):1029–35. 10.1038/ni1006-1029 16985500

[45] Sanjuan MA Dillon CP Tait SW Moshiach S Dorsey F Connell S Toll-like receptor signalling in macrophages links the autophagy pathway to phagocytosis. Nature (2007) 450(7173):1253–7. 10.1038/nature06421 18097414

[46] Chong CS Cao M Wong WW Fischer KP Addison WR Kwon GS Enhancement of T helper type 1 immune responses against hepatitis B virus core antigen by PLGA nanoparticle vaccine delivery. J Control Release (2005) 102(1):85–99. 10.1016/j.jconrel.2004.09.014 15653136

[47] Schlosser E Mueller M Fischer S Basta S Busch DH Gander B TLR ligands and antigen need to be coencapsulated into the same biodegradable microsphere for the generation of potent cytotoxic T lymphocyte responses. Vaccine (2008) 26(13):1626–37. 10.1016/j.vaccine.2008.01.030 18295941

[48] Unanue ER Turk V Neefjes J. Variations in MHC class II antigen processing and presentation in health and disease. Annu Rev Immunol (2016) 34:265–97. 10.1146/annurev-immunol-041015-055420 26907214

[49] De Souza Rebouças J Esparza I Ferrer M Sanz ML Irache JM Gamazo C. Nanoparticulate adjuvants and delivery systems for allergen immunotherapy. J Biomed Biotechnol (2012) 2012:474605. 10.1155/2012/474605 22496608 PMC3303624

[50] Hunter Z McCarthy DP Yap WT Harp CT Getts DR Shea LD A biodegradable nanoparticle platform for the induction of antigen-specific immune tolerance for treatment of autoimmune disease. ACS Nano (2014) 8(3):2148–60. 10.1021/nn405033r 24559284 PMC3990004

[51] Koppelman B Neefjes JJ de Vries JE de Waal Malefyt R. Interleukin-10 down-regulates MHC class II alphabeta peptide complexes at the plasma membrane of monocytes by affecting arrival and recycling. Immunity (1997) 7(6):861–71. 10.1016/S1074-7613(00)80404-5 9430231

[52] Thibodeau J Bourgeois-Daigneault MC Huppé G Tremblay J Aumont A Houde M Interleukin-10-induced MARCH1 mediates intracellular sequestration of MHC class II in monocytes. Eur J Immunol (2008) 38(5):1225–30. 10.1002/eji.200737902 18389477 PMC2759377

[53] Blank F Gerber P Rothen-Rutishauser B Sakulkhu U Salaklang J De Peyer K Biomedical nanoparticles modulate specific CD4+ T cell stimulation by inhibition of antigen processing in dendritic cells. Nanotoxicology (2011) 5(4):606–21. 10.3109/17435390.2010.541293 21231795

[54] Getts DR Martin AJ McCarthy DP Terry RL Hunter ZN Yap WT Microparticles bearing encephalitogenic peptides induce T-cell tolerance and ameliorate experimental autoimmune encephalomyelitis. Nat Biotechnol (2012) 30(12):1217–24. 10.1038/nbt.2434 23159881 PMC3589822

