# Supplementary material for: Reduction of T-Helper Cell Responses to Recall Antigen Mediated by Codelivery with Peptidoglycan via the Intestinal Nanomineral–Antigen Pathway
Source: Front Immunol. 2017 Mar 17;8:284. doi: 10.3389/fimmu.2017.00284 (PMC5355426; doi:10.3389/fimmu.2017.00284)
Supplement: Supplementary file 1 [file Data_Sheet_1.pdf]

Supplementary Material

Reduction of T-helper Cell Responses to Recall Antigen Mediated by Co-delivery with Peptidoglycan via the Intestinal Nanomineral-Antigen

Rachel E Hewitt[1,2*], Jack Robertson[2], Carolin T Haas[2], Laetitia C Pele[2], and Jonathan J Powell[1,2].

[1]Department of Veterinary Medicine, University of Cambridge. Madingley Rd, Cambridge CB3 0ES, UK.

[2]Medical Research Council, Dept. of Mineral Science and Technology, Elsie Widdowson Laboratory, Fulbourn Road, Cambridge CB1 9NL, UK.

*** Correspondence:** Corresponding Author: Rachel.Hewitt@mrc-ewl.cam.ac.uk

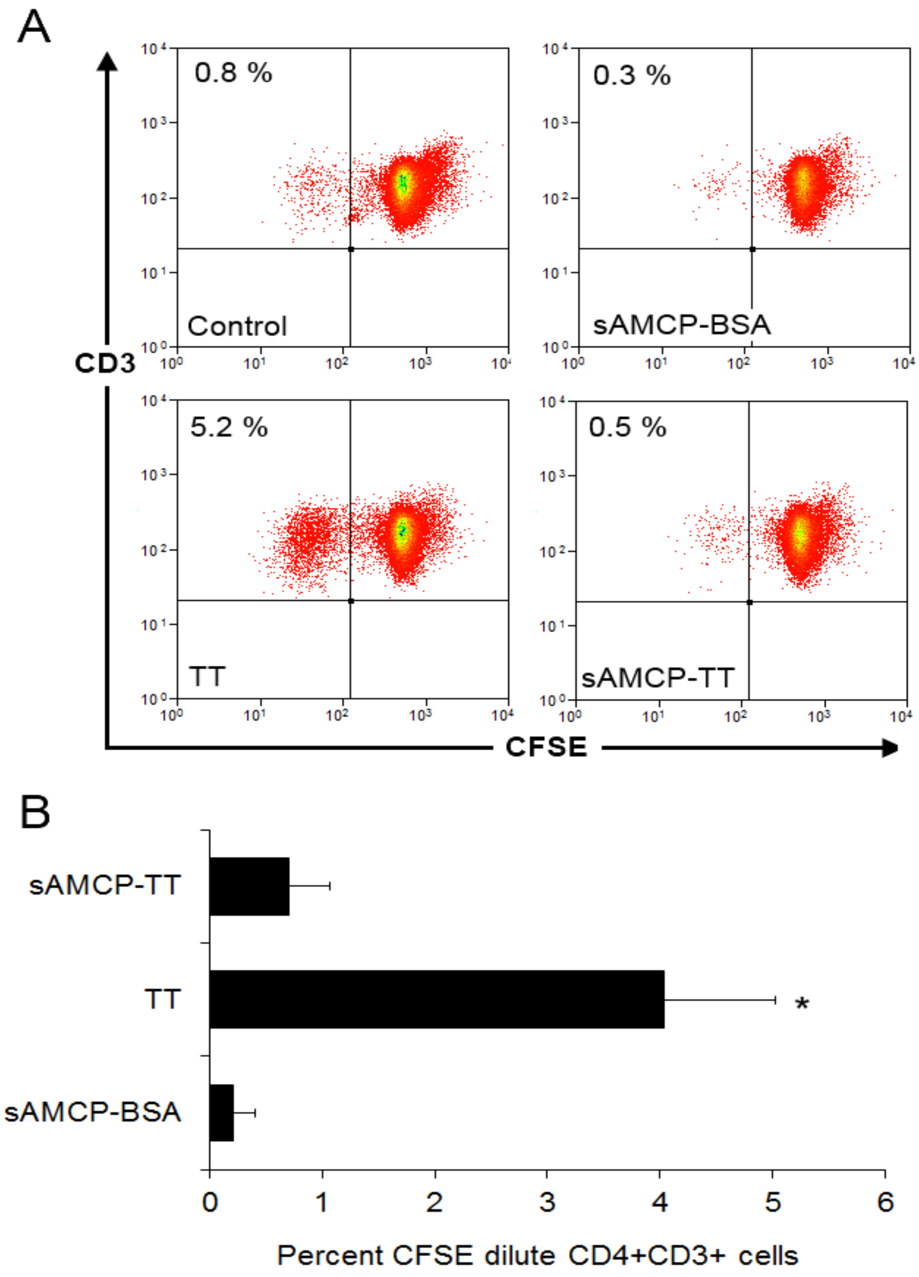

Supplementary Figure 1. Antigen specific CD4^{+} T cell proliferation in response to whole tetanus toxoid in soluble and synthetic AMCP nanomineral form. (**A**) Example flow cytometric plots showing CD4^{+}CD3^{+} dividing cells (CFSE low) in a CFSE proliferation assay; cells within the lymphocyte gate were gated for CD4^{+}CD3^{+} positivity and CD3 versus CFSE was plotted. (**B**) Proliferation of tetanus toxoid (TT) specific CD4^{+}CD3^{+} T cells in PBMC at day 5 in response to soluble tetanus toxoid (TT) and nanomineral TT (sAMCP-TT), (data are mean ± SEM; n = 3 TT responders, * P = 0.04 soluble TT versus sAMCP-TT).

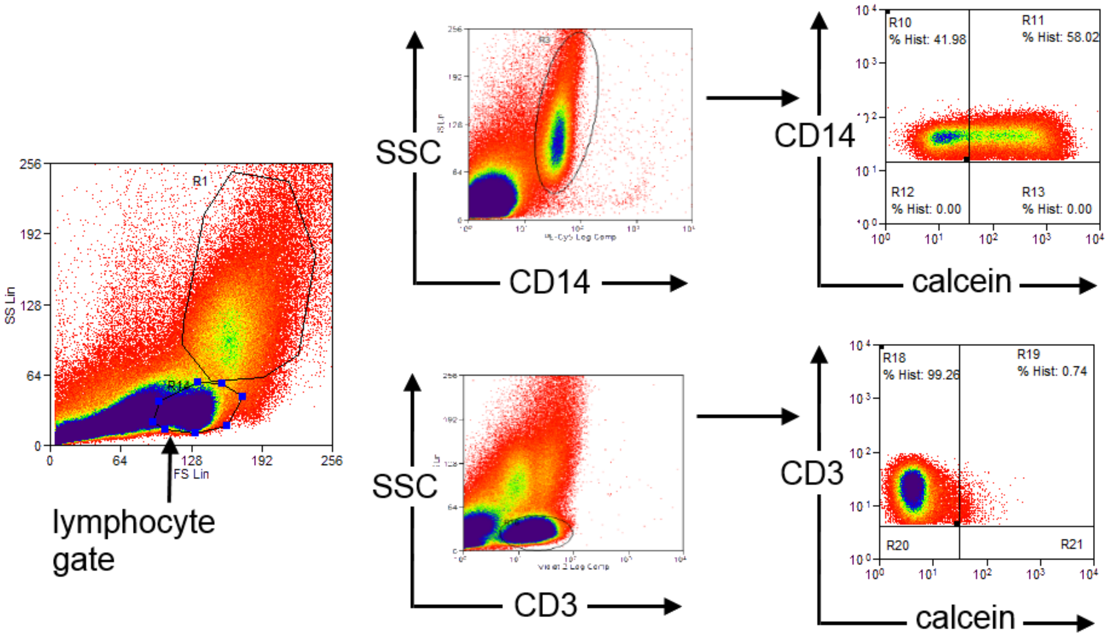

Supplementary Figure 2. Synthetic AMCP nanomineral uptake by monocytes. Example flow cytometric plots showing calcein labelled sAMCP association with $CD14^+$ monocytes. A lymphocyte gate was drawn based on forward versus side scatter profiles and then further gated for CD3 positivity for cells residing in the lymphocyte gate. $CD14^+$ monocytes were gated by plotting side scatter versus $CD14^+$. $CD3^+$ gated lymphocytes and $CD14^+$ gated monocytes were finally plotted against calcein to obtain percentage of $CD14^+$ and $CD3^+$ cells within PBMC positively associating with calcein labelled sAMCP.

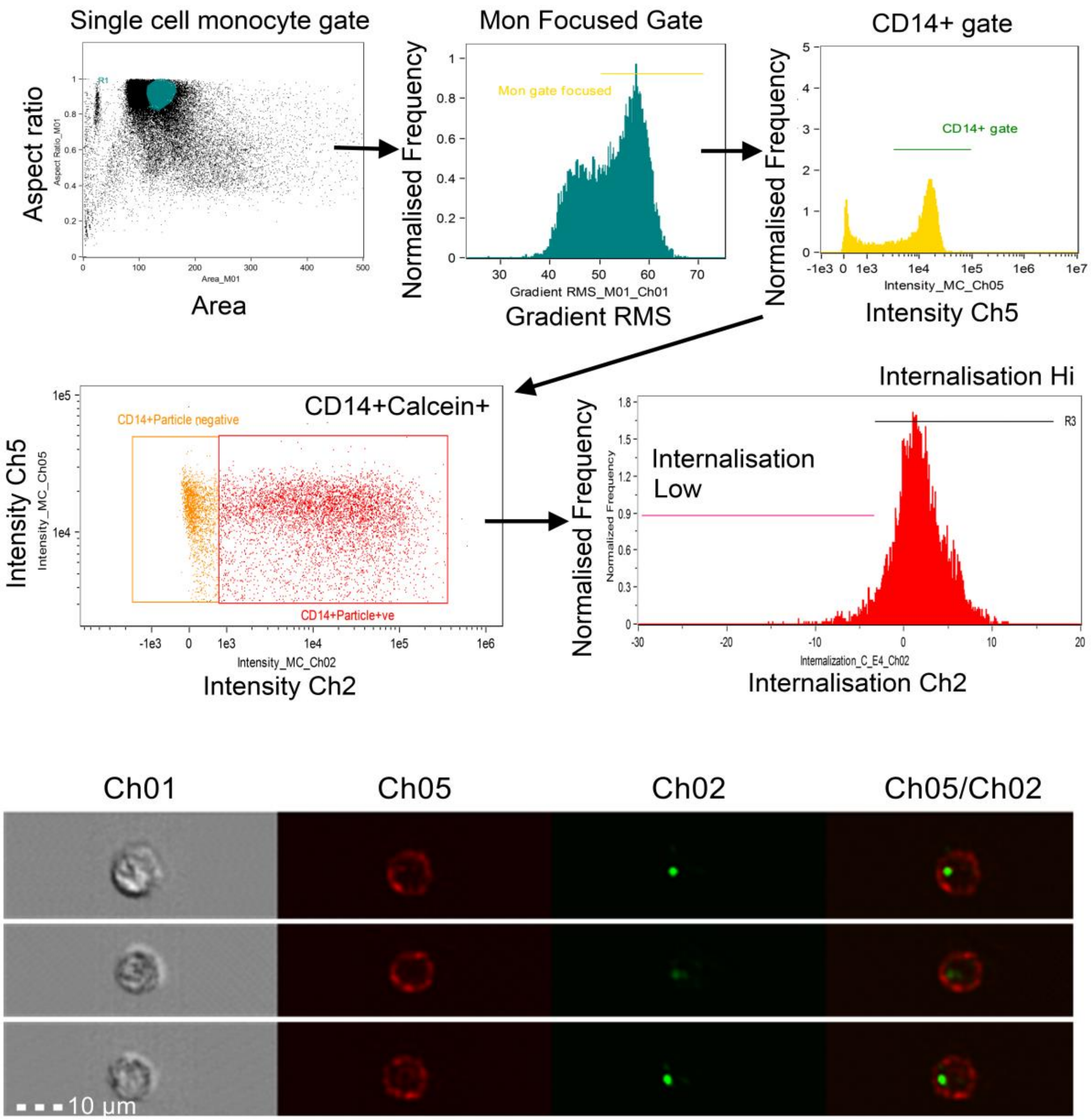

Supplementary Figure 3. Synthetic AMCP nanomineral internalisation by monocytes within PBMC. ImagestreamX analysis of particle internalisation: example plots showing the initial single cell monocyte gate, followed by focused cell gate and subsequent CD14$^+$ gate (top row plots). Calcein positive CD14$^+$ cells were identified by plotting CD14$^+$ intensity (Ch5) versus calcein intensity (Ch2, second row plot) and internalisation scores measured for calcein internalisation (within the cell surface boundary CD14$^+$) for cells residing within the CD14$^+$Calcein$^+$ gate. Examples of internalisation hi cells are shown in the third row (images).

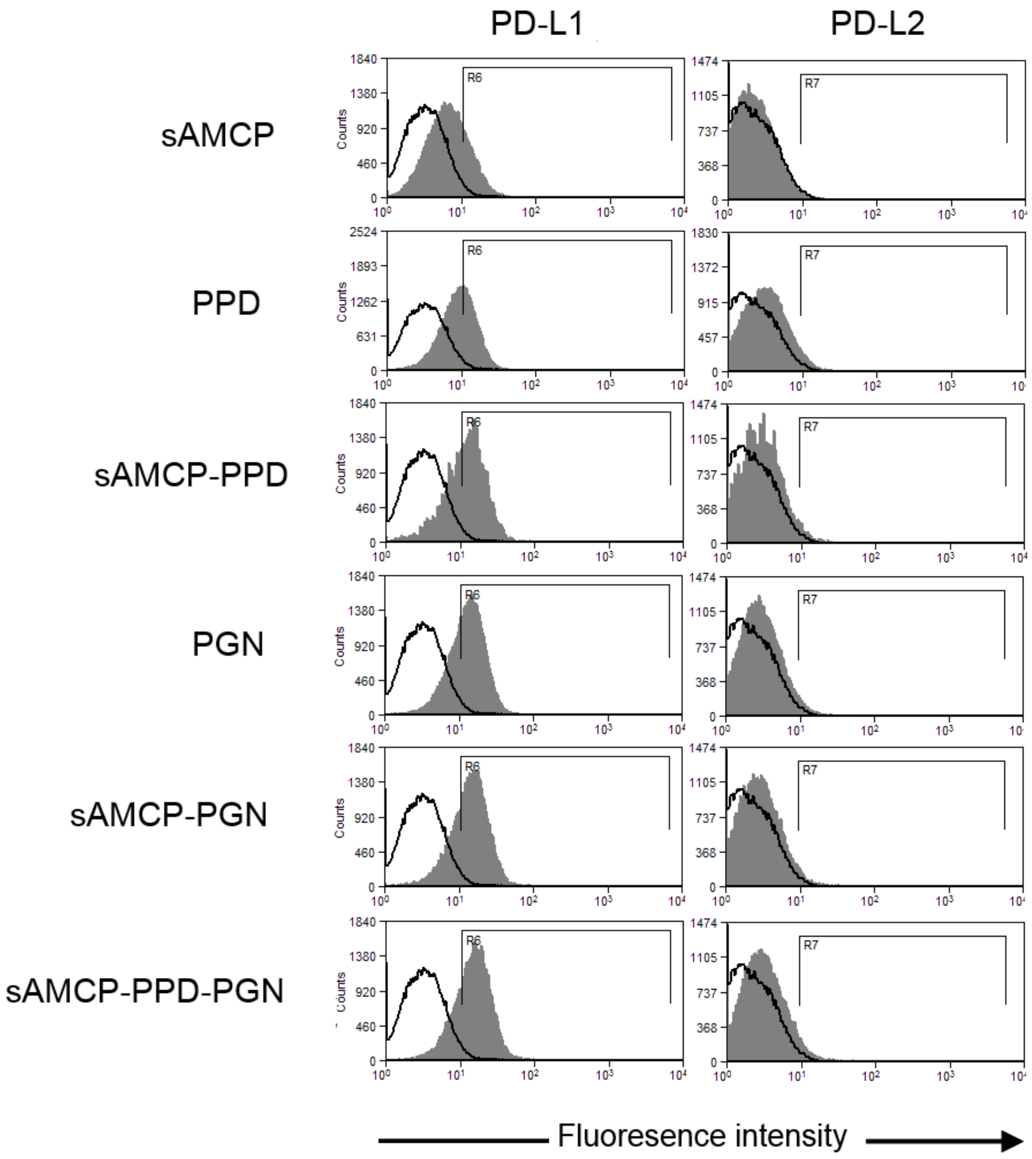

Supplementary Figure 4. APC expression of PD-L1 and PD-L2 in response to synthetic AMCP nanomineral carriage of PGN and PPD – Example flow cytometry plots. Example histogram subtraction plots of pre-gated $CD14^+$ cell surface expression of PD-L1 and PD-L2 from 1 subject's PBMC. Grey shaded area = media only control, black line no fill = response to stimulant indicated.
